# The Impact of a Social Networking Service–Enhanced Smart Care Model on Stage 5 Chronic Kidney Disease: Quasi-Experimental Study

**DOI:** 10.2196/15565

**Published:** 2020-04-14

**Authors:** Feng-Jung Yang, Ying-Hui Hou, Ray-E Chang

**Affiliations:** 1 Institute of Health Policy and Management College of Public Health National Taiwan University Taipei Taiwan; 2 Department of Internal Medicine National Taiwan University Hospital Yun Lin Branch Douliu Taiwan; 3 School of Medicine College of Medicine National Taiwan University Taipei Taiwan; 4 Graduate Institute of Clinical Medicine College of Medicine National Taiwan University Taipei Taiwan; 5 Department of Internal Medicine National Taiwan University Hospital Taipei Taiwan; 6 Department of Medical Genetics National Taiwan University Hospital Taipei Taiwan; 7 Department Health Industry Management School of Healthcare Management Kainan University Taoyuan Taiwan

**Keywords:** chronic kidney disease, stage 5 chronic kidney disease, chronic care model, dialysis initiation, social networking services, social networking, healthcare

## Abstract

**Background:**

Stage 5 chronic kidney disease (CKD) presents a high risk for dialysis initiation and for complications such as uremic encephalopathy, uremic symptoms, gastrointestinal bleeding, and infection. One of the most common barriers to health care for patients with stage 5 CKD is poor continuity of care due to unresolved communication gaps.

**Objective:**

Our aim was to establish a powerful care model that includes the use of a social networking service (SNS) to improve care quality for patients with CKD and safely delay dialysis initiation.

**Methods:**

We used a retrospective cohort of CKD patients aged 20-85 years who received care between 2007 and 2017 to evaluate the efficacy of incorporating an SNS into the health care system. In 2014, author F-JY, a nephrologist at the National Taiwan University Hospital Yunlin Branch, started to use an SNS app to connect with stage 5 CKD patients and their families. In cases of emergency, patients and families could quickly report any condition to F-JY. Using this app, F-JY helped facilitate productive interactions between these patients and the health care system. The intention was to safely delay the initiation of dialysis therapy. We divided patients into four groups: group 1 (G1) included patients at the study hospital during the 2007-2014 period who had contact only with nephrologists other than F-JY; group 2 (G2) included patients who visited F-JY during the 2007-2014 period before he began using the SNS app; group 3 (G3) included patients who visited nephrologists other than F-JY during the 2014-2017 period and had no interactions using the SNS; and group 4 (G4) included patients who visited F-JY during the 2014-2017 period and interacted with him using the SNS app.

**Results:**

We recruited 209 patients with stage 5 CKD who had been enrolled in the study hospital’s CKD program between 2007 and 2017. Each of the four groups initiated dialysis at different times. Before adjusting for baseline estimated glomerular filtration rate (eGFR), the G4 patients had a longer time to dialysis (mean 761.7 days, SD 616.2 days) than the other groups (G1: mean 403.6 days, SD 409.4 days, *P*=.011 for G4 vs G1; G2: 394.8 days, SD 318.8 days, *P*=.04; G3: 369.1 days, SD 330.8 days, *P*=.049). After adjusting for baseline eGFR, G4 had a longer duration for each eGFR drop (mean 84.8 days, SD 65.1 days) than the other groups (G1: mean 43.5 days, SD 45.4 days, *P*=.005; G2: mean 42.5 days, SD 26.5 days, *P*=.03; G3: mean 3.8.7 days, SD 33.5 days, *P*=.002).

**Conclusions:**

The use of an SNS app between patients with stage 5 CKD and their physicians can reduce the communication gap between them and create benefits such as prolonging time-to-dialysis initiation. The role of SNSs and associated care models should be further investigated in a larger population.

## Introduction

### Background

The incidence of end-stage renal disease (ESRD) in Taiwan is very high [1], with an estimated prevalence of 15.46% for all stages of chronic kidney disease (CKD) and 9.06% for CKD stages 3-5 [2]. Research has shown that patients with advanced CKD enrolled in a pre-ESRD pay-for-performance program had lower risks of progressing to ESRD as well as lower risks of mortality and a significantly longer time to initiation of dialysis therapy (430 versus 347 days; *P*<.001) [3]. In a review of 32 articles reporting on 19 trials, earlier initiation of dialysis therapy was not found to reduce mortality compared to later initiation [4]. Economic analyses based on findings from the Initiating Dialysis Early And Late (IDEAL) trial and the US Renal Data System suggest that significant cost savings could be achieved by reversing the trend toward early initiation [5].

The most common barriers to health care reported by patients with CKD include poor continuity of care (eg, seeing a different specialist each visit; 49.3%), inadequate understanding and education about CKD (43.5%), feeling unwell (42.6%), and having trouble maintaining dietary and fluid restrictions (40.1%) [6]. The Chronic Care Model (CCM) is a well-developed and validated framework that illustrates a comprehensive approach to caring for people with chronic illness in a way that supports improved functional and clinical outcomes [7]. The most important aspect of CCM for CKD patients is self-management support, because successful self-management requires CKD patients to know how to monitor their disease, to manage symptoms, to interpret the results of home-monitoring therapies, and to carry out daily treatment plans, including adhering to medication regimens and dietary and fluid restrictions and dealing with side effects.

Surprisingly, clinicians tend to overlook patient access to and use of information and communication technologies (ICTs) to manage their health [8,9]. One study reported that less than 25% of CKD patients obtained information about renal health care from the internet. The ICTs most preferred by their renal health care teams were telephone (56.5%), internet (50%), email (48.3%), and text messages (46%) [9].

However, health care systems and patients are increasingly turning to the internet—including websites as well as social media platforms—for health-related information and support. The US-based National Kidney Foundation, for example, designed a comprehensive and user-friendly digital ecosystem that contains content relevant to each audience and helps promote prompt interactions between CKD patients and their health care providers as envisioned by the CCM [10]. The ecosystem received high satisfaction scores (88%) on the ForeSee survey, a customer satisfaction survey administered on the US National Institute of Diabetes and Digestive and Kidney Diseases websites [10]. Results from a paper by Gee et al [11] showed that chronic care needs to reform to incorporate ICT tools. They concluded that (1) eHealth (electronic health) education has a critical role in self-care; (2) eHealth support should be put into the community, and patients should be empowered with the benefits of the e-community (electronic community) or virtual communities; and (3) productive technology-based interactions ensure feedback loop between the patient and the provider [11].

A randomized controlled trial found no difference in mean estimated glomerular filtration rate (eGFR) at baseline and the number of patients who progressed renal replacement regardless of use of a standardized self-management program (where patient education, telephone-based support, and support groups were delivered by a multidisciplinary team of management nurses, dietitians, peers, and volunteer) [12]. In another study, the University of Pittsburgh Medical Center instituted an impressive patient- and family-initiated rapid response system called Condition Help based out of a hospital [13]. Safety issues could be identified and prevented through the Condition Help system, although the majority (83.4%) of calls involved nonsafety issues. In one study looking at a cohort of Hispanic patients with CKD, lower patient-physician interaction scores were independently associated with a higher risk of hospitalization but not with incidence of ESRD or death [14]. 

Further testing of ICT interventions to improve self-management is necessary. Despite the potential benefits of ICTs for health care, few studies have addressed the usage and preferences regarding these technologies among patients with chronic diseases such as CKD and ESRD [10].

### Objectives

A new model of physician-patient interaction, the eHealth Enhanced Chronic Care Model, was discussed by Gee et al [11] (Multimedia Appendix 1). Following this model, our study aimed to establish the value of a new kind of connection between patients and the hospital where they are receiving care—a connection that uses a social networking service (SNS) to encourage proactive action. Our study explores a smart care model with SNS to determine whether it improves how patients and providers connect across digital platforms and to advance our understanding of how an SNS might be able to improve health-related outcomes for patients with CKD.

## Methods

### Study Population

In 2002, Taiwan’s health care system, known as National Health Insurance, launched the Project of Integrated Care for CKD, a nationwide pre-ESRD pay-for-performance program providing more comprehensive care to patients with CKD (stages 3-5). The National Taiwan University Hospital (NTUH) Yunlin Branch, a regional teaching hospital in southern Taiwan, joined this program in 2004 and prospectively enrolled patients with CKD (stages 3-5) who were willing to participate. Patients were diagnosed with CKD according to the criteria of the National Kidney Foundation’s Kidney Disease Outcomes Quality Initiative (KDOQI) clinical practice guidelines [15] and received follow-up care at an outpatient department. Biochemical profiles including serum creatinine, blood urea nitrogen (BUN), and the spot urine protein to creatinine ratio (UPCR) were measured at least every 12 weeks. Our study followed all enrolled patients until initiation of long-term renal replacement therapy (hemodialysis, peritoneal dialysis, or transplantation) or until December 31, 2017, whichever occurred first.

#### Patient Selection

For our study, we selected a retrospective cohort of patients who had stage 5 CKD when they enrolled in the Project of Integrated Care for CKD. To further ensure that study patients were regularly treated for CKD, eligible patients were required to have had at least two outpatient visits within 3 months of their first date of diagnosis. In our final analysis, we included 209 patients aged 20 to 85 years with stage 5 CKD who had enrolled in the program and remained in it between 2007 and 2017.

#### Patient Grouping

Author F-JY is a nephrologist at the teaching hospital where this study took place. In 2015, he started using an SNS app to connect with his patients with stage 5 CKD and their families. In cases of emergency, patients and families could quickly report any condition to F-JY through this app. In this way, F-JY helped promote productive interactions and prompt responses between patients with stage 5 CKD and the health care system. In addition, family members and patients could exchange messages and photos with their health care providers via this platform. No medical decisions were made on this platform; if any risks emerged, clinic appointments were made. All nephrologists at the hospital acted in accordance with the guidelines of the KDOQI and the pay-for-performance program.

To examine the effect of the SNS intervention on care for patients with stage 5 CKD, we employed a quasi-experimental (single group pre-post) study design. We divided patients into four groups. Group 1 (G1) included patients who had contact only with hospital nephrologists other than F-JY during the 2007-2014 period; group 2 (G2) included patients who visited F-JY during the 2007-2014 period before he began using the SNS app; group 3 (G3) included patients who visited nephrologists other than F-JY during the 2014-2017 period and had no SNS interactions; and group 4 (G4) included patients who visited F-JY during the 2014-2017 period and used the SNS. The number of patients per group is shown in Multimedia Appendix 2.

#### Social Networking Service

The SNS used by F-JY was Line, a mobile app operated by Naver Corporation. Users can use texts, images, video, and audio for contact, and have free voice conversations and video conferences at any time. In Taiwan, the Line app has become increasingly popular since 2014 [16].

### Measurement of Kidney Function

A serum creatinine level of <15 ml/min/1.73 m2 was used to define the baseline eGFR and establish the patient’s CKD stage as 5 at enrollment. eGFR was calculated with the Modification of Diet in Renal Disease equation:

eGFR = 175 × creatinine^–1.154^ × age^–0.203^ × 1.212 (if black) × 0.742 (if female)

The value of eGFR was recorded by every 3 months until dialysis. The rate of decline in daily eGFR was defined as baseline eGFR divided by time to dialysis in days.

### Data Collection

Blood samples were collected before every clinic visit. Hematological and biochemical tests were conducted in the central laboratory of the hospital. Baseline comorbidities and clinical laboratory data were recorded by taking medical histories and conducting detailed chart reviews. All medical histories were recorded, including diabetes, hypertension, and cardiovascular disease (defined as coronary artery disease, myocardial infarction, stroke, and heart failure), after reviewing the electronic medical records.

### Statistical Analyses

Continuous variables were reported as means and standard deviations, or medians with interquartile ranges if distributions were skewed. Categorical variables, such as cardiovascular risk factors (eg, hypercholesterolemia, hypertension, and smoking), were reported as frequencies and percentages.

Baseline characteristics, including demographics (age and gender), laboratory data (sodium, potassium, calcium, phosphate, BUN, creatinine, and albumin), and presence of comorbid disease (coronary artery disease, congestive heart failure, hypertension, diabetes mellitus, and malignancy) were compared between the groups. Baseline patient characteristics were compared using chi-square tests for categorical variables and the Mann-Whitney *U* test for continuous variables.

We performed multiple linear regression analysis to evaluate the associations between time to dialysis, duration of each eGFR drop, and daily eGFR decline rate with baseline covariables. We combined difference in differences with matching on pretreatment outcomes to address nonparallel trends between the treatment and control groups. The covariables included care model, age, diabetes mellitus (yes vs no), albumin, and hemoglobin. A multiple logistic regression analysis was performed to determine the factors associated with time to dialysis, duration of each eGFR drop, and daily eGFR decline rate. Statistical analyses were performed using SPSS Version 25 (IBM) and Prism 7.0d (GraphPad).

### Ethics Statement

The study was approved by the NTUH ethical review board (NTUH 201901030RINB and 201903005RINA). To maintain confidentiality, all data sets in the study were pseudonymized, and personal IDs, birth dates, and names were encrypted. This deidentification process was supervised by NTUH’s Institutional Review Board (IRB), which verified the anonymity of data analysis performed in this study. Because the data were analyzed anonymously and in accordance with IRB guidelines, informed consent was not obtained from the study participants. All research procedures followed the directives of the Declaration of Helsinki.

## Results

### Baseline Measurements

We investigated the differences in demographics and clinical and laboratory data among the four study groups. There were no differences between groups in terms of gender, age, diabetes, hypertension, hemoglobin (g/dL), baseline eGFR, BUN (mg/dL), creatinine (mg/dL), sodium (mmol/L), potassium (mmol/L), calcium (mg/dL), phosphorus (mg/dL), or uric acid (mg/dL). As shown in Table 1, patients in the four groups did show differences in time to dialysis, albumin, and calcium levels, all of which were statistically significant.

**Table 1:** Baseline demographic, clinical, and laboratory measurements by group.

Measurement^a^	Group 1^b^		Group 2^b^			Group 3^b^		Group 4^b^		Total			*P* value^c^
	Value	n	Value	n	Value		n	Value	n	Value	N		
Male, n (%)	39 (44)	88	18 (55)	33	40 (56)		71	8 (47)	17	105 (50)	209	.46	
Age (years), mean (SD)	66.8 (13.0)	88	67.2 (12.7)	33	66.9 (13.9)		71	63.1 (10.2)	17	66.6 (13.0)	209	.72	
Diabetes mellitus (yes), n (%)	45 (51)	88	18 (48)	33	37 (52)		71	10 (59)	17	109 (52)	209	.92	
Hypertension (yes), n (%)	58 (66)	88	20 (61)	33	51 (72)		71	12 (71)	17	140 (67)	209	.69	
Albumin (g/dL), mean (SD)	3.7 (0.4)	86	4.0 (0.5)	33	3.6 (0.5)		71	3.8 (0.5)	17	3.7 (0.5)	207	*.005*	
Hemoglobin (g/dL), mean (SD)	9.0 (1.5)	87	9.5 (1.6)	33	9.4 (1.4)		71	8.8 (1.5)	17	9.2 (1.5)	208	.26	
eGFR (mL/min/1.73 m^2^), mean (SD)	9.2 (2.9)	88	9.1 (3.1)	33	9.8 (3.0)		71	9.1 (2.0)	17	9.4 (2.9)	209	.44	
Time to dialysis (days), mean (SD)	403.6 (409.4)	88	394.8 (318.8)	33	369.1 (330.8)		71	761.7 (616.2)	17	419.6 (403.0)	209	*.003*	
BUN (mg/dL), mean (SD)	86.4 (24.3)	87	93.7 (39.8)	33	94.9 (35.4)		71	88.6 (31.5)	17	90.6 (31.7)	208	.37	
Creatinine (mg/dL), mean (SD)	9.4 (3.9)	88	9.7 (3.9)	33	8.8 (3.5)		71	9.7 (3.8)	17	9.3 (3.8)	209	.66	
Sodium (mmol/L), mean (SD)	137.5 (4.3)	77	137.7 (4.6)	31	136.9 (4.7)		45	137.1 (3.8)	13	137.3 (4.4)	166	.84	
Potassium (mmol/L), mean (SD)	4.7 (0.7)	88	4.5 (0.7)	33	4.6 (0.7)		71	4.9 (0.6)	17	4.6 (0.7)	209	.29	
Calcium (mg/dL), mean (SD)	8.8 (0.9)	87	9.1 (1.0)	33	8.3 (1.4)		71	8.7 (0.7)	17	8.7 (1.1)	208	*.001*	
Phosphorus (mg/dL), mean (SD)	5.9 (1.7)	87	5.9 (1.6)	33	5.8 (1.5)		71	6.2 (1.4)	17	5.9 (1.6)	208	.80	
Uric acid (mg/dL), mean (SD)	8.4 (1.9)	80	8.4 (2.1)	32	7.5 (2.8)		47	7.1 (2.2)	13	8.1 (2.2)	172	.05	

^a^Measurements are for patients with stage 5 CKD at time of enrollment.

^b^Groups are stratified by physician and care model. Group 1: patients who had contact only with hospital nephrologists other than author F-JY, 2007-2014; group 2: patients who visited F-JY before he began using the SNS app, 2007-2014; group 3: patients who visited nephrologists other than F-JY, 2014-2017 (no SNS interactions); group 4: patients who visited F-JY and used the SNS app, 2014-2017.

^c^*P* values express differences in data between groups. *P* values less than .05 are marked in italics.

### Age and Time to Dialysis

Aging—but not age—presented a risk for dialysis initiation. As shown in Table 2, age did not increase with daily eGFR decline rate in late-enrolled patients with stage 5 CKD. Decline in daily eGFR, albumin, hemoglobin, care model, and eGFR at enrollment were not associated with age, but age showed a positive association with eGFR at the last outpatient visit (*r*=.136, *P*=.049). In addition, time to dialysis was associated positively with age, albumin, and eGFR at enrollment but negatively with diabetes.

**Table 2:** Pearson correlations (*r*) between age and other risk factors, and time to dialysis and other risk factors, among enrolled patients with stage 5 chronic kidney disease.

Variable		*r*	*P* value^a^
**Age**			
	Daily eGFR^b^ decline rate	−.065	.35
	Albumin	.063	.37
	Hemoglobin	−.026	.71
	Diabetes mellitus (yes)	−.046	.51
	Care model	−.058	.41
	eGFR at enrollment	.081	.25
	eGFR at last outpatient visit	.136	*.049*
**Time to dialysis**			
	Age	*.166*	*.02*
	Diabetes mellitus (yes)	−*.183*	*.008*
	Hemoglobin	.016	.82
	Albumin	*.254*	*<.001*
	Blood urea nitrogen	.094	.18
	eGFR at enrollment	*.268*	*<.001*
	Creatinine	.104	.14
	Sodium	.080	.31
	Potassium	−.081	.24
	Calcium	.113	.11
	Phosphorus	−.015	.83

^a^*P* values less than .05 are marked in italics.

^b^eGRF: estimated glomerular filtration rate.

### Time to Dialysis and Duration of Each eGFR Drop

**Figure 1:**
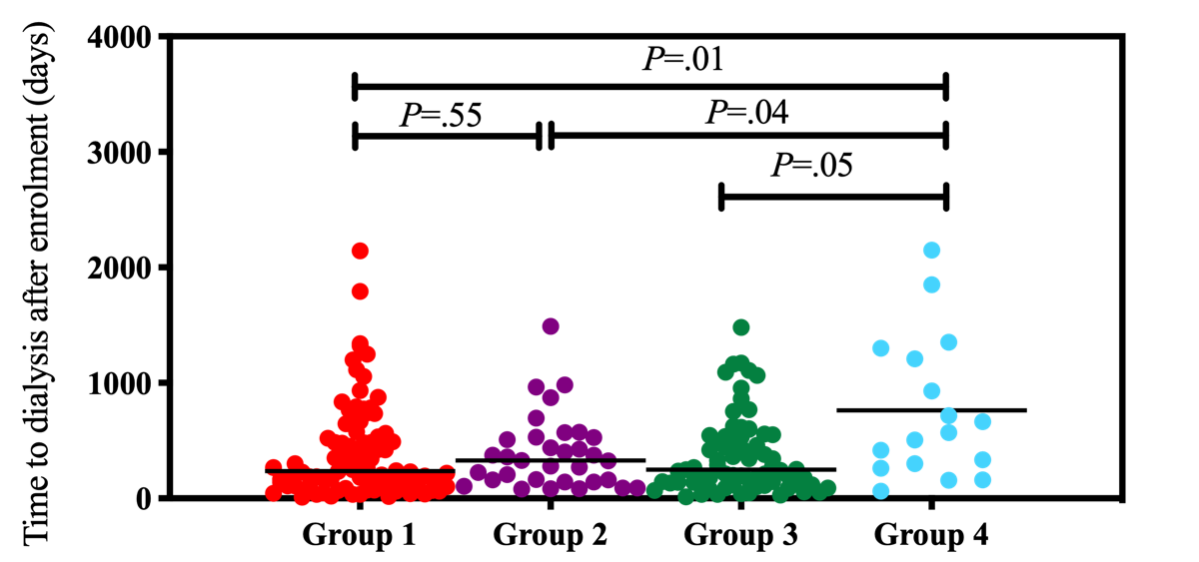
Trends in time to dialysis were compared between the four groups. The lines indicate median values. Statistical calculation of *P* values was performed using the nonparametric Mann-Whitney *U* test.

**Figure 2:**
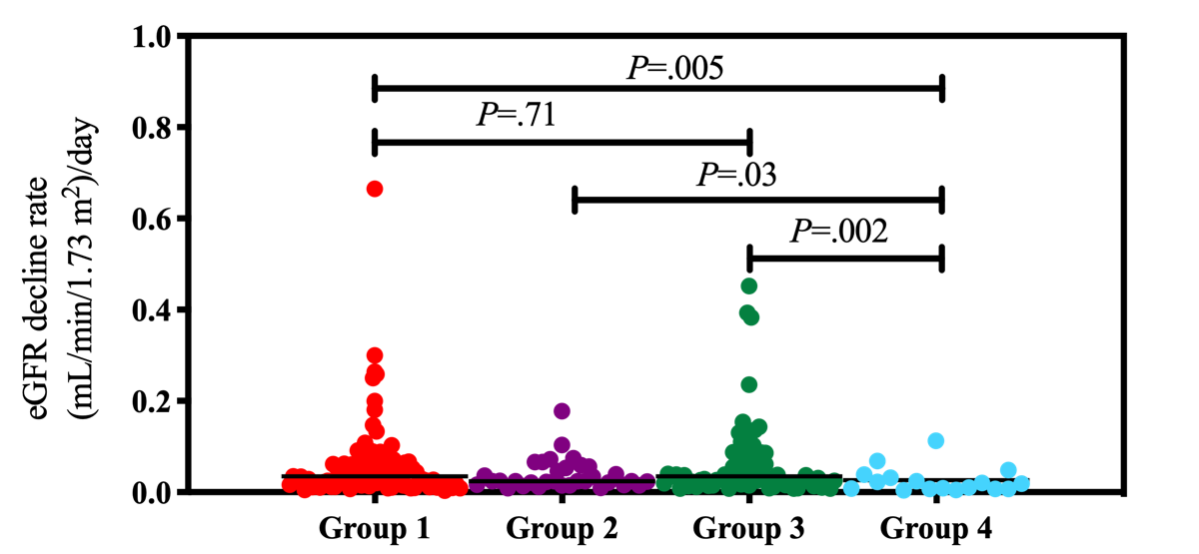
Trends in the decline of daily estimated glomerular filtration rate (eGFR; [mL/min/1.73 m^2^]/day) were compared between the four groups. The lines indicate median values. Statistical calculation of *P* values was performed using the nonparametric Mann-Whitney *U* test.

**Figure 3:**
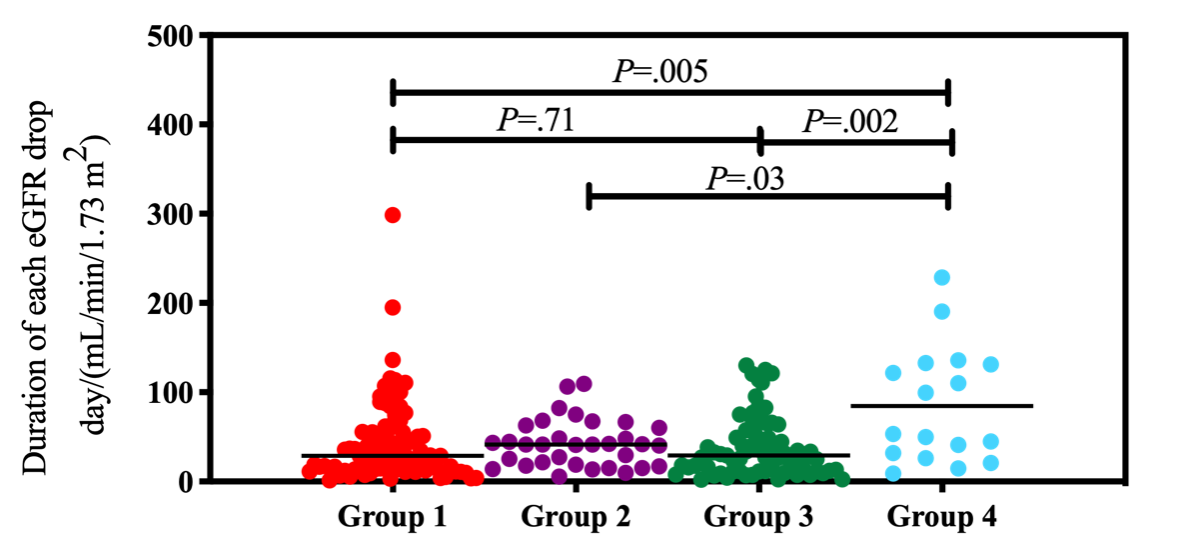
Durations of each drop in estimated glomerular filtration rate (EGFR; day/[mL/min/1.73 m^2^]) were compared between the four groups. The lines indicate median values. Statistical calculation of *P* values was performed using the nonparametric Mann-Whitney *U* test.

Time to dialysis differed among the four groups of patients. Before adjusting for baseline eGFR, patients in G4 had a longer time to dialysis initiation (mean 761.7 days, SD 616.2 days) than patients in the other groups (G1: mean 403.6 days, SD 409.4 days, *P*=.011; G2: mean 394.8 days, SD 318.8 days, *P*=.04; G3: mean 369.1 days, SD 330.8 days, *P*=.049; Figure 1). Each patient showed differences in renal function at baseline. As shown in Table 3, we adjusted time to dialysis according to baseline eGFR. The duration of each eGFR drop is defined by time to dialysis (days) divided by the baseline eGFR (mL/min/1.73 m^2^). G4 had longer durations for each eGFR drop (mean 84.8 days, SD 65.1 days) compared to the other groups (G1: mean 43.5 days, SD 45.4 days, *P*=.005; G2: mean 42.5 days, SD 26.5 days, *P*=.03; G3: mean 38.7 days, SD 33.5 days, *P*=.002; Figures 2 and 3).

**Table 3:** Comparison of clinical and laboratory measurements by group, adjusted for baseline estimated glomerular filtration rate (eGFR).

Measurement^a^	Group 1	Group 2	Group 3	Group 4	*P* value^b^
Age (years), mean (SD)	66.8 (13.0)	67.2 (12.7)	66.9 (13.9)	63.1 (10.2)	.72
Diabetes mellitus (yes), n (%)	45 (51)	16 (48)	37 (52)	10 (59)	.92
Albumin (g/dL), mean (SD)	3.7 (0.4)	4.0 (0.5)	3.6 (0.5)	3.8 (0.5)	*.005*
Hemoglobin (g/dL), mean (SD)	9.0 (1.5)	9.5 (1.6)	9.4 (1.4)	8.8 (1.5)	.26
eGFR (mL/min/1.73 m^2^), mean (SD)	9.2 (2.9)	9.1 (3.1)	9.8 (3.0)	9.1 (2.0)	.44
Time to dialysis (days), mean (SD)	403.6 (409.4)	394.8 (318.8)	369.1 (330.8)	761.7 (616.2)	.*003*
Duration of each eGFR drop, (days/[mL/min/1.73 m^2^]), mean (SD)	43.5 (45.4)	42.5 (26.5)	38.7 (33.5)	84.8 (65.1)	*.010*

^a^Measurements are for patients with stage 5 CKD at time of enrollment.

^b^*P* values less than .05 are marked in italics.

### Difference in Differences in the Care Model

Finally, we tested the independent association of the new care model and time to dialysis, and combined difference in differences with matching on pretreatment outcomes to address nonparallel trends between groups. We measured two factors: factor A was the individual physician factor (ie, “team” versus F-JY, where “team” refers to the involvement of any nephrologist other than F-JY); and factor B refers to the intervention phase (ie, the control phase, 2007-2014, versus the experimental phase, 2014-2017). Multivariable-adjusted logistic regression models, including age, diabetes mellitus, albumin, and hemoglobin, were used to investigate the independent association between factor A, factor B, and time to dialysis.

As shown in Table 4, the interaction of factor A with factor B was independently associated with time to dialysis in regression models after adjustment for age, diabetes mellitus, albumin, and hemoglobin. We found that the new care model driven by SNS had increased time to dialysis to 459.38 days. Similar results were found when the duration of each eGFR drop was used in the regression model instead of time to dialysis (Table 5). The SNS care model increased the duration of each eGFR drop to 52.7 days. In addition, in Table 6, we showed results in comparison with a reference group of adult patients less than 50 years of age. Multivariable-adjusted logistic regression models, including three other age groups (50-59 years, 60-69 years, and >70 years), diabetes mellitus, gender, albumin, and hemoglobin, were used to investigate the independent association between factor A (team vs F-JY), factor B (control vs experimental), and time to dialysis. Using difference in differences, our new care model had an increased time to dialysis of approximately 417.6 days.

**Table 4:** Associations between time to dialysis and care model using difference in differences^a^.

Variable	Unstandardized coefficients		Standardized coefficients			
	B	SE	Beta	*t*	*P* value^b^	95% CI
(Constant)	−569.77	292.84	—^c^	−1.95	.053	−1147.27 to 7.72
Age	5.60	2.02	0.18	2.77	*.006*	1.62 to 9.58
Diabetes	−117.53	54.44	−0.15	−2.16	*.03*	−224.89 to −10.17
Hemoglobin	10.51	17.57	0.039	0.60	.55	−24.14 to 45.16
Albumin	155.07	56.87	0.19	2.73	*.007*	42.92 to 267.22
Factor A: team vs author F-JY	−67.08	77.94	−0.07	−0.86	.39	−220.78 to 86.63
Factor B: control vs experimental	−30.32	60.79	−0.04	−0.50	.62	−150.19 to 89.56
Factor A × Factor B	459.38	128.31	0.31	3.58	*<.001*	206.36 to 712.40

^a^Multivariable-adjusted logistic regression models, including age, diabetes mellitus, albumin, and hemoglobin, were used to investigate the independent association between factor A, factor B, and time to dialysis.

^b^*P* values less than .05 are marked in italics.

^c^Not applicable.

**Table 5:** Associations between the duration of each drop in estimated glomerular filtration rate and the care model using difference in differences^a^.

Variable	Unstandardized coefficients		Standardized coefficients			
	B	SE	Beta	*t*	*P* value^b^	95% CI
(Constant)	−50.55	30.81	—^c^	−1.64	.10	−111.30 to 10.21
Age	0.43	0.21	0.13	2.03	*.044*	0.01 to 0.85
Diabetes	−14.54	5.73	−0.17	−2.54	*.012*	−25.83 to −3.24
Hemoglobin	1.18	1.85	0.04	0.64	.53	−2.47 to 4.82
Albumin	16.98	5.98	0.20	2.84	*.005*	5.18 to 28.78
Factor A: team vs author F-JY	−7.17	8.20	−0.07	−0.88	.38	−23.34 to 9.00
Factor B: control vs experimental	−4.16	6.40	−0.05	−0.65	.52	−16.77 to 8.45
Factor A × factor B	52.71	13.50	0.34	3.91	*<.001*	26.09 to 79.33

^a^Multivariable-adjusted logistic regression models, including age, diabetes mellitus, albumin, and hemoglobin, were used to investigate the independent association between factor A, factor B, and duration of each eGFR drop.

^b^*P* values less than .05 are marked in italics.

^c^Not applicable.

**Table 6:** Associations between time to dialysis and care model using difference in differences compared with the reference group (age <50 years)^a^.

Variable	Unstandardized coefficients		Standardized coefficients			
	B	SE	Beta	*t*	*P* value^b^	95% CI
(Constant)	−158.05	268.87	—^c^	−0.59	.56	−688.32 to 372.22
Age (50-59.9 years)	−30.66	95.51	−0.03	−0.32	.75	−219.03 to 157.70
Age (60-69.9 years)	−14.67	92.97	−0.02	−0.16	.88	−198.02 to 168.69
Age (≥70 years)	88.57	84.02	0.11	1.05	.29	−77.15 to 254.28
Gender	−176.07	52.94	−0.22	−3.33	*.001*	−280.47 to −71.67
Diabetes	−107.57	53.68	−0.13	−2.00	*.046*	−213.44 to −1.70
Hemoglobin	9.94	17.22	0.04	0.58	.56	−24.02 to 43.90
Albumin	155.03	55.78	0.19	2.78	*.006*	45.03 to 265.03
Factor A: team vs author F-JY	−32.56	77.01	−0.04	−0.42	.67	−184.44 to 119.32
Factor B: control vs experimental	−5.31	59.98	−0.007	−0.09	.93	−123.60 to 112.98
Factor A × factor B	417.61	126.71	0.29	3.30	*.001*	167.72 to 667.50

^a^Multivariable-adjusted logistic regression models, including age, diabetes mellitus, albumin, and hemoglobin, were used to investigate the independent association between factor A (team vs F-JY), factor B (control vs experimental), and duration of each eGFR drop. The dependent variable was time to dialysis.

^b^*P* values less than .05 are marked in italics.

^c^Not applicable.

## Discussion

### Principal Findings

This study is significant in that it is the first study to combine an SNS with standard care for patients with stage 5 CKD. Other studies of SNSs and CKD recruited patients with CKD stages 3-4 only or patients with an unknown CKD stage. In this study, the physician, not the case manager, played the central role in integrated care.

Our study demonstrates the effectiveness of a new care model that includes use of an SNS for patients with stage 5 CKD and their physicians. After introducing the model, patients using the SNS care model saw benefits such as a longer time to dialysis, a longer duration of each eGFR drop, and a daily decline in eGFR, perhaps because they gained more real-time mental health–related and other support from their physicians.

### Comparison With Prior Work

The IDEAL trial, a randomized controlled trial, showed a median time to initiation of dialysis of 1.80 months (95% CI 1.60 to 2.23) in its early-start group and 7.40 months (95% CI 6.23 to 8.27) in its late-start group, but showed no significant difference between the groups in terms of adverse events (cardiovascular events, infections, or complications of dialysis) [17]. This trial has demonstrated it can be safe to wait for lower eGFR levels or specific symptoms before beginning dialysis.

Razzaghi and Afshar [18] reported on the four key components of the physician-patient healing relationship: (1) valuing the patient as a person, (2) effective management of the power imbalance between the physician and the patient, (3) commitment, and (4) competence and character of the physician. They additionally stated that the three necessary relational elements of physician-patient relationship are trust, peace and hope, and acknowledgment [18].

Patient adherence to treatment is highly influenced by the quality of communication patients receive during medical care [19]. Physicians must be trained in efficient communication to enhance this adherence. The physician-patient relationship, therefore, plays a central role in patient health-related outcomes. The components of this relationship may impact the patient’s experience of the health system.

Our study addressed the potential of a primary physician–led care model with an SNS in delaying dialysis initiation for patients with stage 5 CKD. This SNS-integrated care practice showed a significant effect in the intervention group compared with the control group in slowing the daily reduction in eGFR, and there was significant improvement in time to initiation of dialysis among patients in the intervention group compared with patients in the control group (with an imbalance between groups resulting from their relatively small sample size). This is a quasi-experimental design that suggests that SNSs in primary care can increase patient awareness, delay dialysis initiation, and provide patients with more mental health support from their physician.

### Future Directions

To our knowledge, this is the first study of SNS that took place in a real-world primary care practice that measured outcomes for patients with stage 5 CKD longitudinally. To confirm the usefulness of the SNS care model, more data and objective results regarding efficacy are needed. The role of SNSs and associated care models should be further investigated in a larger population. Observational studies, clinical trials, systematic reviews, and meta-analyses should aim to further establish the role of digital health technologies in patient care.

### Limitations

This study is retrospective to review the quality of care with SNS in a teaching hospital. The sample size is relatively small and only few physicians are involved in the study.

### Conclusions

Use of SNSs by patients with stage 5 CKD and their physicians could resolve important communication gaps and create better conditions for treatment of CKD. In our study, we demonstrated that use of an SNS in a physician-led care model was associated with a significant delay in dialysis initiation for stage 5 CKD patients. SNSs can act as a communication bridge and assist in improving the connection between the health care system and the community. Patient adherence is highly correlated to communication between the medical caregiver and the patient. Better communication skills improve patient adherence and outcomes [19]. Consistent with other works, we found that high-quality communication and interpersonal support can improve care quality.

## Appendix

Multimedia Appendix 1 The eHealth enhanced Chronic Care Model.

Multimedia Appendix 2 Grouping of patients with stage 5 chronic kidney disease by physician and care model (with or without social networking service).

## Abbreviations

BUN: blood urea nitrogen
CCM: Chronic Care Model
CKD: chronic kidney disease
e-community: electronic community
eGFR: estimated glomerular filtration rate
eHealth: electronic health
ESRD: end-stage renal disease
G1-4: groups 1 to 4
ICT: information and communication technology
IDEAL: Initiating Dialysis Early And Late
IRB: Institutional Review Board
KDOQI: Kidney Disease Outcomes Quality Initiative
NTUH: National Taiwan University Hospital
SNS: social networking service
UPCR: urine protein to creatinine ratio
