# Supplementary material for: The Impact of a Social Networking Service–Enhanced Smart Care Model on Stage 5 Chronic Kidney Disease: Quasi-Experimental Study
Source: J Med Internet Res. 2020 Apr 14;22(4):e15565. doi: 10.2196/15565 (PMC7189249; doi:10.2196/15565)
Supplement: Multimedia Appendix 1 [file jmir_v22i4e15565_app1.docx]

**Multimedia Appendix 1**

**The eHealth Enhanced Chronic Care Model.**

**Adapted from Gee PM, Greenwood DA, Paterniti DA, Ward D, Miller LM,** **The eHealth Enhanced Chronic Care Model: a theory derivation approach, J Med Internet Res 2015;17(4):e86.**
