# Supplementary material for: The Impact of a Social Networking Service–Enhanced Smart Care Model on Stage 5 Chronic Kidney Disease: Quasi-Experimental Study
Source: J Med Internet Res. 2020 Apr 14;22(4):e15565. doi: 10.2196/15565 (PMC7189249; doi:10.2196/15565)
Supplement: Multimedia Appendix 2 [file jmir_v22i4e15565_app2.docx]

**Multimedia Appendix 2**

**Grouping of patients with stage 5 CKD by physician and care model (with or without SNS)**
